# Molecular detection and species identification of *Alexandrium* (Dinophyceae) causing harmful algal blooms along the Chilean coastline

**DOI:** 10.1093/aobpla/pls033

**Published:** 2012-12-18

**Authors:** Ana Jedlicki, Gonzalo Fernández, Marcela Astorga, Pablo Oyarzún, Jorge E. Toro, Jorge M. Navarro, Víctor Martínez

**Affiliations:** 1 FAVET-INBIOGEN, Faculty of Veterinary Sciences, University of Chile, Santa Rosa No 11.735 (PO 8820808), Santiago, Chile; 2 Aquaculture Institute, Austral University of Chile, Los Pinos (PO. 1327), Puerto Montt, Chile; 3 Limnological and Marine Science Institute, Austral University of Chile (PO. 567), Valdivia, Chile

## Abstract

**Background and aims:**

On the basis of morphological evidence, the species involved in South American Pacific coast harmful algal blooms (HABs) has been traditionally recognized as *Alexandrium catenella* (Dinophyceae). However, these observations have not been confirmed using evidence based on genomic sequence variability. Our principal objective was to accurately determine the species of *Alexandrium* involved in local HABs in order to implement a real-time polymerase chain reaction (PCR) assay for its rapid and easy detection on filter-feeding shellfish, such as mussels.

**Methodology:**

For species-specific determination, the intergenic spacer 1 (ITS1), 5.8S subunit, ITS2 and the hypervariable genomic regions D1–D5 of the large ribosomal subunit of local strains were sequenced and compared with two data sets of other *Alexandrium* sequences. Species-specific primers were used to amplify signature sequences within the genomic DNA of the studied species by conventional and real-time PCR.

**Principal results:**

Phylogenetic analysis determined that the Chilean strain falls into Group I of the *tamarensis* complex. Our results support the allocation of the Chilean *Alexandrium* species as a toxic *Alexandrium tamarense* rather than *A. catenella*, as currently defined*.* Once local species were determined to belong to Group I of the *tamarensis* complex, a highly sensitive and accurate real-time PCR procedure was developed to detect dinoflagellate presence in *Mytilus* spp. (Bivalvia) samples after being fed (challenged) *in vitro* with the Chilean *Alexandrium* strain. The results show that real-time PCR is useful to detect *Alexandrium* intake in filter-feeding molluscs.

**Conclusions:**

It has been shown that the classification of local *Alexandrium* using morphological evidence is not very accurate. Molecular methods enabled the HAB dinoflagellate species of the Chilean coast to be assigned as *A. tamarense* rather than *A. catenella.* Real-time PCR analysis based on *A. tamarense* primers allowed the detection of dinoflagellate DNA in *Mytilus* spp. samples exposed to this alga. Through the specific assignment of dinoflagellate species involved in HABs, more reliable preventive policies can be implemented.

## Abstract (precis)

Misleading morphological observations assigned *Alexandrium catenella* as local dinoflagellate responsible for HABs in Southern Chilean coasts. Our work based on molecular methods found that local *Alexandrium* belongs to group I of the *tamarensis* complex composed mainly of *A. tamarense.*

## Introduction

Harmful algal blooms (HABs) occur throughout the world and are known for their negative economic and sanitary impacts (Anderson 2009). Of particular concern are the paralytic shellfish toxins produced mainly by bloom-forming dinoflagellates in the genus *Alexandrium.* Over the past few decades, *Alexandrium* blooms have extended, covering new territories. In this sense, expansion of dinoflagellate species could be explained by ocean currents, human-induced mechanisms such as water from ballasts and global warming, climate adaptation and colonization of newly generated niches (Anderson 1989). On the other hand, it is also possible that the increasing number of blooms reported today is the result of a worldwide effort to implement new techniques to detect and prevent their negative effects (Anderson 2007). Blooms of different *Alexandrium* species have been reported from Japan (Kodama 2010), northwestern Mediterranean Sea (Vila *et al.* 2001), Australia (Hallegraeff 1998), Caribbean Sea, off the Venezuelan coast (Halstead and Schantz 1984), Brazil (Persich *et al.* 2006), along the American Pacific coasts, from Alaska to the Strait of Magellan (Suárez *et al.* 2002; Glibert *et al.* 2005; Hernández *et al.* 2005), and from north Atlantic coasts from the Gulf of St Lawrence to North Carolina (Anderson *et al.* 1994). Compared with the numerous studies describing Australian, North American and Japanese *Alexandrium* ribotypes and morphotypes, relatively few works describe their South American counterparts from the South Pacific (Lilly 2003).

Historically, *Alexandrium* species were described based on microscopic observations of morphological features including plate patterns, cell size and shape, and secondary characteristics such as chain formation. Unfortunately, these morphological traits have often proven insufficient for identifying species, leading to confusion concerning the distribution, ecology and toxicity within this genus (Lilly *et al.* 2005). When morphological features are questionable for taxonomic identification, they must be combined with molecular data for accurate species definition (Hansen *et al.* 2003).

Sequence variation analyses have been accepted to be a valid methodology for an accurate species description. This is even clearer in the *Alexandrium* genus, which has been partially reclassified on the basis of molecular genetic data, as the taxonomic value of only morphological characters proved to be insufficient for the *tamarensis* complex (Leaw *et al.* 2005). A good example is the taxonomic trait ‘presence or absence of the ventral pore’, used to discriminate between *A. affine* and *Alexandrium tamarense.* This trait would not be deemed useful for species identification considering that it is homoplastic (Leaw *et al.* 2005).

Using the classical species definition for lineage formation (Mayr 1982), Lilly *et al*. (2007) recognize *Alexandrium tamarense* (Lebour) Balech, *Alexandrium catenella* (Whedon and Kofoi) Balech and *Alexandrium fundyense* Balech as different species. On the other hand, phylogenies of *Alexandrium* species have been established based on genomic sequences of the large and small subunits of ribosomal DNA (LSU and SSU rDNA, respectively) (Guillou *et al.* 2002; Usup *et al.* 2002; John *et al*. 2003, 2005; Murray *et al.* 2005; Rogers *et al.* 2006). Of these sequences, the D1/D2 region of the LSU rDNA has been proved to be the most suited for discrimination of closely related *Alexandrium* species (Ki and Han 2007). Thus, this hypervariable region has been proposed as a suitable candidate to discriminate between species with similar fidelity as Cytochrome Oxidase I gene (Sonnenberg *et al.* 2007). Scholin and Anderson (1994, 1996) and Scholin *et al.* (1994, 1995), based on DNA sequencing of the divergent D1/D2 LSU rDNA region and restriction fragment length polymorphism (RFLP) analysis of the small subunit rDNA genes, consider that they could be strains of the same species, naming them the *tamarensis* complex. Using these results as a starting point, Lilly *et al.* (2007) further established the *tamarensis* complex as a valid cluster, derived from a phylogenetic analysis comparing more than 126 different *Alexandrium* D1/D2 region sequences. Their detailed examination revealed the presence of five clades, defined as: Group I (North American), Group II (Mediterranean), Group III (Western European), Group IV (Temperate Asian) and Group V (Tasmanian).

In this study, we sequenced local *Alexandrium* intergenic spacer 1 (ITS1), 5.8S rDNA, ITS2 and D1–D5 hypervariable LSU rDNA regions in order to incorporate molecular data that could help define more clearly the *Alexandrium* species responsible for HABs in Chilean coasts. This is considering that the local South American Pacific *Alexandrium* species were classified as *A. catenella* (Muñoz 1985), mainly based on morphological traits, but without a further assessment of sequence variation. In this sense, species-specific assignment allows the implementation of a polymerase chain reaction (PCR) assay for accurate monitoring along Chilean coasts in order to prevent public health hazards and economic losses.

## Methods

### Cell cultures

Three different clonal *Alexandrium* cell cultures (ACC01, ACC02, ACC07) were kindly provided by Professor Benjamín Suárez from the Laboratorio de Toxinas Marinas, Universidad de Chile. These were collected from Canal Costa in Aysén region, Chile (45°37′60 S, 73°32′60 W), between April 1994 and March 1995, and maintained in f/2 medium (Guillard 1975) at 12 °C under a 16 : 8 h light:dark cycle and 60 μmol m^−2^ s^−1^ photon flux density. These three strains belong to the Chilean algae repository collection used in various national and international studies (Córdoba and Müller 2002; Amaro *et al*. 2005; Lilly *et al.* 2007; Montoya *et al.* 2010).

### DNA purification from cell cultures

Cells for analysis (100 mL) were collected from each clonal culture at mid-logarithmic phase and centrifuged at 3000 *g* for 5 min at 4 °C. The supernatant was removed, the pellet resuspended in 500 μL of Milli-Q water, and transferred to a 1.5-mL microfuge tube. Microfuge tubes were placed in liquid nitrogen for 30 s and the cells subsequently disrupted for 1 min using an Axygen polypropylene pestle (PES-15-B-SI, Union City, CA, USA). Genomic DNA was then extracted from the pellet using the DNeasy Plant Mini Kit (Qiagen*,* Hilden, Germany) according to the manufacturer's instructions.

### Determination of *Alexandrium* DNA concentration and quality

DNA concentration was measured using a fluorometer (Qubit, Sunnyvale, CA, USA) together with the Qubit dsDNA BR Assay Kit (Invitrogen, Eugene, OR, USA). The quality was evaluated based on its integrity by comparison with a 23-kb band of λ-HindIII ladder (Invitrogen, USA) in 1 % agarose (Ultrapure, Invitrogen, Barcelona, Spain) gel electrophoresis stained with ethidium bromide.

### PCR amplification and sequence analysis

In order to determine the species of the three isolates, the ITS1-D5 rDNA was amplified, sequenced and then aligned with sequences from GenBank (Ki and Han 2007). All amplifications were carried out in duplicate with 1× PCR buffer, 20–50 ng of genomic DNA template, 3 mM MgCl_2,_ 100 µM each dNTP, 0.1 µM each primer and 0.4 U of recombinant Taq DNA polymerase (MBI Fermentas, Vilnius, Lithuania) in a 10-µL reaction volume. Polymerase chain reaction primer sequences for LSU rDNA and optimized annealing temperatures are specified in Table 1. Polymerase chain reaction parameters were: 95 °C for 5 min; 35 cycles of denaturation at 95 °C for 30 s, annealing for 30 s, extension at 72 °C for variable time spans which depended upon the size of the amplifying fragment (1000 bases min^−1^); and a final extension at 72 °C for 5 min. Reactions were run on a MaxyGene Gradient thermocycler (Axygen). Five microlitres of PCR products were analysed by 2 % agarose (Invitrogen, USA) gel electrophoresis according to standard methods. rDNA PCR amplification products from clones ACC01, ACC02 and ACC07 were purified from gels using the MinElute Gel Extraction Kit (Qiagen) following the manufacturer's instructions and directly sequenced in an ABI PRISM 3100. An electropherogram base quality assignment algorithm, phred (Ewing and Green 1998; Ewing *et al.* 1998), was used to re-analyse the sequenced fragment of all strains in order to determine intragenomic polymorphic sites.

**Table 1:** Primer sequences used in amplifying the ITS1-D5 region in *Alexandrium* species.

Primer name	Nucleotide sequence 5′ to 3′	Annealing temperature (°C)	Reference
catF	cctcagtgagattgtagtgc	Between 45 and 65	Hosoi-Tanabe and Sako (2005)
catR	gtgcaaaggtaatcaaatgtcc		
tamF	tgcttggtgggagtgttgca	66	Hosoi-Tanabe and Sako (2005)
tamR	taagtccaaggaaggaagcatc		
tamF-1	tgagggaaatatgaaaaggac	TD 58–48 (−0.5 )	This study
tamR-1	attcggcaggtgagttgtta		
tamF-2	gaaggagaagtcgtaacaagg	TD 58–48 (−0.5 )	This study
tamR-2	caatgccaaggagtgtgac		

The annealing temperature for each primer and the study from which the sequences were obtained are listed.

Additional confirmation of species identification was achieved by amplifying the extracted DNA using four microsatellite primers specific to *A. catenella* (Nagai *et al.* 2005; Table 2) or *A. tamarense* (Alpermann *et al.* 2006; Table 2). The amplification conditions were the same as those provided in the original publications describing the assays.

**Table 2:** Primer sequences used in amplifying species-specific microsatellite genomic regions in *A. catenella* and *A. tamarense*.

Primer	Nucleotide sequence 5′ to 3′	Annealing temperature (°C)	Species	Reference
Acat02-F	caagtgaactaaatccgct	60	*A. catenella*	Nagai *et al.* (2005)
Acat02-R	aaaacggaatgtttatgtgc			
Acat16-F	tgtctttcttcctgcctgcctt	60	*A. catenella*	Nagai *et al.* (2005)
Acat16-R	ttcaccccagcgaagccattatg			
Acat20-F	aggagaaaagtgatgcatctcagcaa	60	*A. catenella*	Nagai *et al.* (2005)
Acat20-R	aatcctgtggatgatggaaggtactg			
Acat44-F	tgccccataagggttcttccaga	60	*A. catenella*	Nagai *et al.* (2005)
Acat44-R	gacagtggtattgcaaacccaacggat			
ATB1-F	cgcctgctcgagaaaaga	53	*A. tamarense*	Alpermann *et al.* (2006)
ATB1-R	ttgggggacagttgagtttc			
ATB8-F	cagggtagccgatcaaacac	TD 61–54 (−0.3)	*A. tamarense*	Alpermann *et al.* (2006)
ATB8-R	cttccatcgccttgcatact			
ATD8-F	caacactggaagcgtgctaa	TD 61–54 (−0.3)	*A. tamarense*	Alpermann *et al.* (2006)
ATD8-R	cccatgcgctacctcttaca			
ATF11-F	agcagcgcggcgggagatt	TD 68.5–61 (−0.3)	*A. tamarense*	Alpermann *et al.* (2006)
ATF11-R	acctgcggctgcgacacgact			

The annealing temperature for each primer, the species and the study from which the sequences were obtained are listed.

TD, touchdown.

### Collection, analysis and association of sequences

All selected *Alexandrium* sequences were obtained using the keywords ‘Alexandrium LSU rDNA’ or ‘Alexandrium 28S in the GenBank database (**http://www.ncbi.nlm.nih.gov**) [see ADDITIONAL INFORMATION]. For a detailed species-specific analysis, two data sets were generated. The first was composed of 81 unique sequences at least 641 bp long, covering the D1/D2 region of the LSU rDNA. This group incorporated 79 *Alexandrium* genus species (including the local strain), and two *Prorocentrum micans* strains that were used as outgroups. The second set had 18 sequences at least 1776 bp long, of which 16 corresponded to *tamarensis* complex (including the local strain), one to *A. minutum* and one to *A. affine*; the last two were used as outgroups. For both data sets, alignments were carried out in the ClustalX2 V2.0 (Larkin *et al.* 2007) graphical platform.

### Substitution model and associated parameter estimation

jModeltest (Posada 2008) was used to find the best substitution model and associated parameters for phylogenetic analysis in both data sets using the Akaike (Hirotugu 1974) and Bayesian (Schwarz 1978) information criteria.

### Phylogenetic analysis

Bayesian analysis was implemented with MrBayes V3.2 (Ronquist *et al.* 2012) for the first and second data sets, and was carried out with 1 500 000 runs, with five separate initial trees, with the Markov chain Monte Carlo (MCMC) process set to four chains and 25 % of initial trees discarded as ‘burn-in’. Within each chain, samples were obtained every 100 iterations, and the values of the average deviation of split frequencies (AVSF) and potential scale reduction factor (PSRF) were obtained. These values were used to evaluate convergence of the generated trees. Additionally, maximum likelihood analysis was carried out in PhyML V3.0 (Guindon and Gascuel 2003) in order to further support taxon assignment. Analysis was started with a random tree sample (Subtree pruning and regrafting method) and 1000 bootstrap replicate runs.

Figtree V1.3.1 (Andrew Rambaut. FigTree v1.3.1 2006–2009. **http://tree.bio.ed.ac.uk/software/figtree**) was used for a graphical visualization and representation of PhyML and MrBayes output trees.

In order to estimate the nucleotide differences within the *tamarensis* complex in each data set, the values for the average number of differences per pair of sequences aligned within each group and the number of parsimonious informative sites were calculated in the MEGA 5.05 program (Tamura *et al*. 2011).

### *Alexandrium* DNA detection in challenged *Mytilus* samples by real-time PCR

An *in situ* experimental protocol for dinoflagellate challenge was developed in order to implement it subsequently for *Alexandrium* detection in filter-feeding shellfish and water columns. The experiments were performed in quadruplicate using four experimental aquaria of 15 L, containing five mussels each (one mussel from each aquarium for each of the 5 days was used for DNA extraction).

Individuals of the edible mussel *Mytilus* were transported to the laboratory, where they were acclimatized for 1 week at 14 °C and seawater salinity of 30 practical salinity units. During this period, mussels were continuously fed with the microalga *Isochrysis galbana* at 1.5 mg L^−1^ using a peristaltic pump and providing constant aeration. The seawater was changed every 48 h. Following acclimation, the mussels were exposed to a contaminated diet (1.7–2.0 mg L^−1^; dry weight) containing 50 % toxic dinoflagellate *Alexandrium* strain ACC02 and 50 % *I. galbana* (by weight) for a period of 12 days, followed by a detoxification period of 15 days where they were fed with *I. galbana*. Every day the aquaria received an amount of food representing 2 % of the dry body weight of the experimental mussels (Shafee 1976), delivered continuously using a Masterflex 7519-05 peristaltic pump at the temperature and salinity cited above. One mussel from each replicate aquarium was taken on Days 2, 3, 4 and 5 of the toxic feeding cycle and on Day 15 of the detoxification cycle. Animals were immediately processed for purification of gill DNA.

**Fig. 1:**
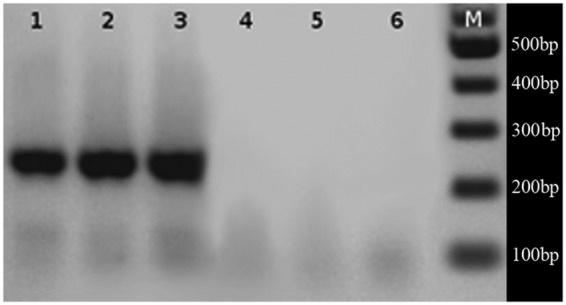
**Amplification of local *Alexandrium* strains ACC01, ACC02 and ACC07 using species*-*specific primers; *A. tamarense* (lanes 1, 2 and 3) and *A. catenella* (lanes 4, 5 and 6).** M = 100-bp DNA size marker. Species-specific amplification in the rDNA region using *A. catenella* and *A. tamarense* primers were carried out in a MaxiGene Gradiente thermocycler (Axygen) in 1× PCR buffer, 20–50 ng of genomic DNA template, 3 mM MgCl_2,_ 100 µM each dNTP, 0.1 µM each primer and 0.4 U of TopTaq DNA polymerase (Fermentas) in a 10-µL reaction volume. Five microlitres of each PCR product were analysed in a 2 % agarose gel. A Fermentas GeneRuller™ 100-bp DNA ladder was used for size estimation of amplified fragments.

To determine the possibility of detecting *Alexandrium* DNA in challenged mussel tissue, we used real-time PCR. Experiments were run in triplicate, for each sample, on an Eco real-time PCR System (Illumina, San Diego, CA, USA) using Quantace SensiMix HRM^tm^ kit (Bioline, London, UK). Reaction conditions were: 1 × SensiMix HRM buffer, 0.6 μL of EvaGreen dye, 0.5 μM primers tamF and tamR (Hosoi-Tanabe and Sako 2005), and 100 ng of challenged *Mytilu*s spp. gill DNA in a 10-μL final reaction volume. The PCR protocol cycling was: a 10-min initial activation step at 94 °C, followed by 40 cycles of 94 °C for 30 s, 55.3 °C for 30 s and 72 °C for 30 s. A PCR amplification product of 235 bp (Fig. 1) obtained with tamF and tamR was purified and sequenced in order to corroborate specificity.

### DNA purification from challenged *Mytilus* gill tissue

Mussels were randomly taken from each of the four aquaria (replicates) on each day of sampling. Animals were dissected alive and 30 g of drained gill tissue were used as the starting material for DNA purification with DNeasy Blood & Tissue Kit (Qiagen) according to the manufacturer's protocol. Each sample of purified DNA was stored at −20 °C. As explained below, the gill was considered as a useful source of *Alexandrium* DNA as particulate materials, such as unicellular organisms, tend to accumulate in this organ (Jørgensen 1996; Riisgard *et al*. 1996) and other tissues such as the hepatopancreas do not provide DNA with the integrity needed for this type of study.

## Results

### PCR amplification of microsatellite genomic regions

**Fig. 2:**
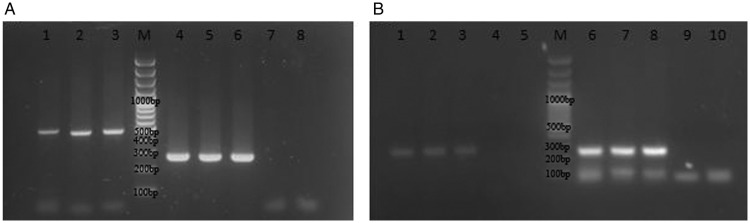
***Alexandrium tamarense* microsatellite amplifications of the three local strains with (A) specific primers ATB8 (lanes 1, 2 and 3) and ATD8 (lanes 4, 5 and 6).** Lanes 7 and 8 correspond to controls with primers ATB8 without DNA. (B) Specific amplification with primers ATB1 (lanes 1, 2 and 3) and ATF11 (lanes 6, 7 and 8). Lanes 4–5 and 9–10 are controls without DNA for primer sets ATB1 and ATF11, respectively. M = 100-bp DNA size marker. Species-specific microsatellite amplifications using *A. catenella* and *A. tamarense* primers were carried out in a MaxiGene Gradiente thermocycler (Axygen) in 1× PCR buffer, 20–50 ng of genomic DNA template, 3 mM MgCl_2,_ 100 µM each dNTP, 0.1 µM each primer and 0.4 U of TopTaq DNA polymerase (Fermentas) in a 10-µL reaction volume. Five microlitres of each PCR product were analysed in a 2 % agarose gel. A Fermentas GeneRuller^TM^ 100-bp DNA ladder was used for size estimation of amplified fragments.

The results of the species-specific PCR of eight microsatellite genomic regions were consistent in all three isolates, amplifying only those directed towards *A. tamarense* and not to *A. catenella* (Fig. 2). No changes could be observed with the *A. catenella* set of primers despite the numerous protocol modifications of PCR conditions.

### Analysis based on ITS1-D5 LSU rDNA sequences

#### Sequence analysis and evaluation

Electropherogram profiles from the ITS1-D5 region of the rDNA of strains ACC01, ACC02 and ACC03 were analysed with the phred algorithm in order to discard the presence of pseudogenes or intragenomic rDNA polymorphisms (IRP). Only bases with scores over 30 (sequencing error probability 1/1000) were considered for further analysis. As no nucleotide differences were obtained for this region between the three local *Alexandrium* strains, only one sequence, Ach01, was used as a representative of them (NCBI accession no. JN657223).

#### Substitution model and associated parameter evaluation

Analysis by jModeltest estimated that the GTR + Γ was the best substitution model for later Bayesian and maximum likelihood analysis. If a given model was not an option in MrBayes, the following least restrictive model was used (e.g. GTR).

#### Phylogenetic analysis

**Fig. 3:**
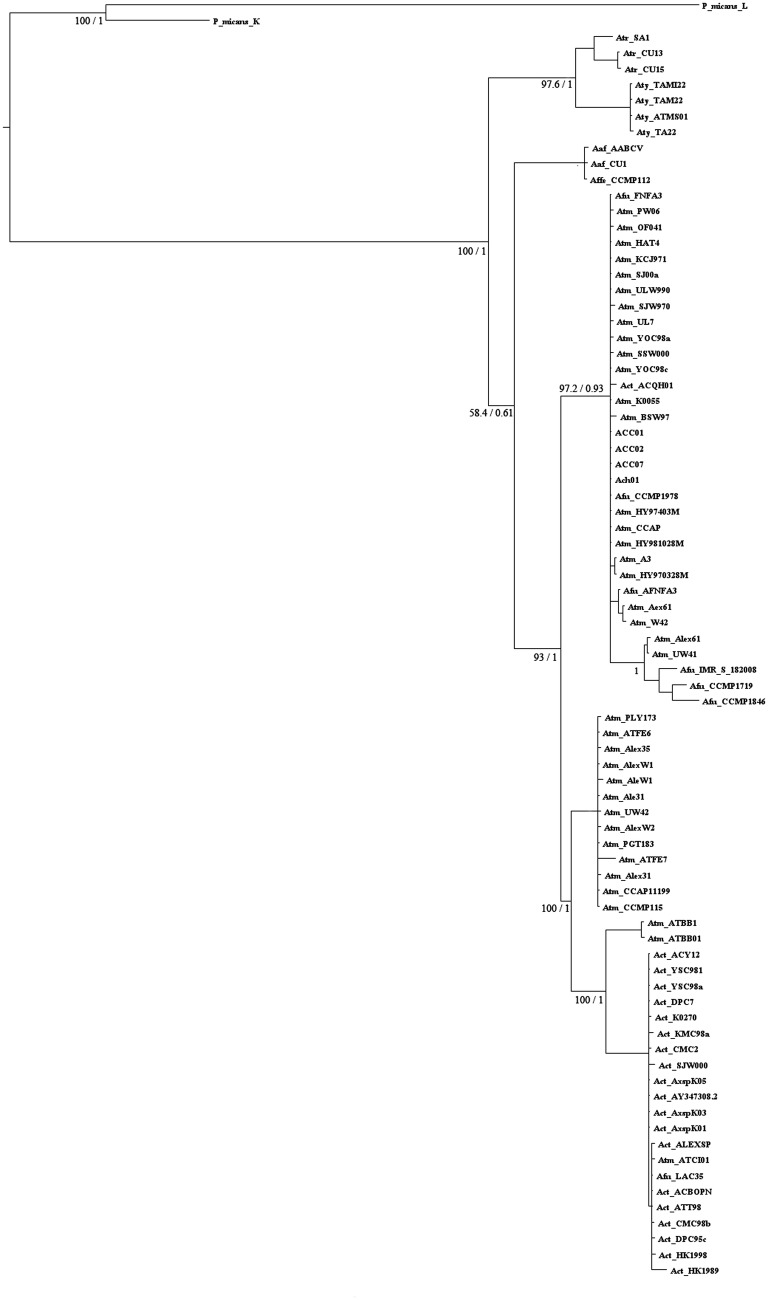
**Phylogenetic tree of 80 *Alexandrium* species and strains (Atm, *A. tamarense*; Act, *A. catenella*; Afu, *A. fundyense*; Amn, *A. minutum*; Aaf, *A. affine*; Atr, *A. tropicale*; Aty, *A. tamiyanavichii*; *P. micans*, *Prorocentrum micans*) and local *Alexandrium* species Ach01.** Sequences were obtained from the GenBank database using the keywords ‘Alexandrium LSU rDNA’ or ‘Alexandrium 28S’. Phylogenetic trees were generated for an alignment of 81 sequences of 641 bp in the D1/D2 region through Bayesian inference in MrBayes V3.2 and maximum likelihood (ML) in PhyML. Bayesian analysis was carried out with 1 500 000 runs, with five separate initial trees. Convergence was checked through PSRF and AVSF. FigTree V1.3.1 was used as a visual representation of output trees. For ML analysis these were carried out with 1000 bootstrap replicates.

Using 81 sequences of length 641 bp, all aligning in the same D1/D2 LSU rDNA region, from different species of the genus *Alexandrium* and two strains of *P. micans*, the phylogenetic distribution of local *Alexandrium* strains could be estimated (Fig. 3). Convergence of Bayesian trees was evaluated through AVSF and PSRF. Values were less than 0.01 and ∼1 ±0.005, respectively, suggesting that the distribution reached a stationary phase in both data sets. Additionally, bootstrap values and the logarithm of the likelihood score of the optimal tree (−4229.43342) extracted by maximum likelihood analysis were indicative of a precise tree. The results indicate that the local *Alexandrium* strain is in Group I, dominated by *A. tamarense*, and is grouped with other previously sequenced *Alexandrium* strains from Chilean waters (ACC01, ACC02 and ACC07; Fig. 3).

**Fig. 4:**
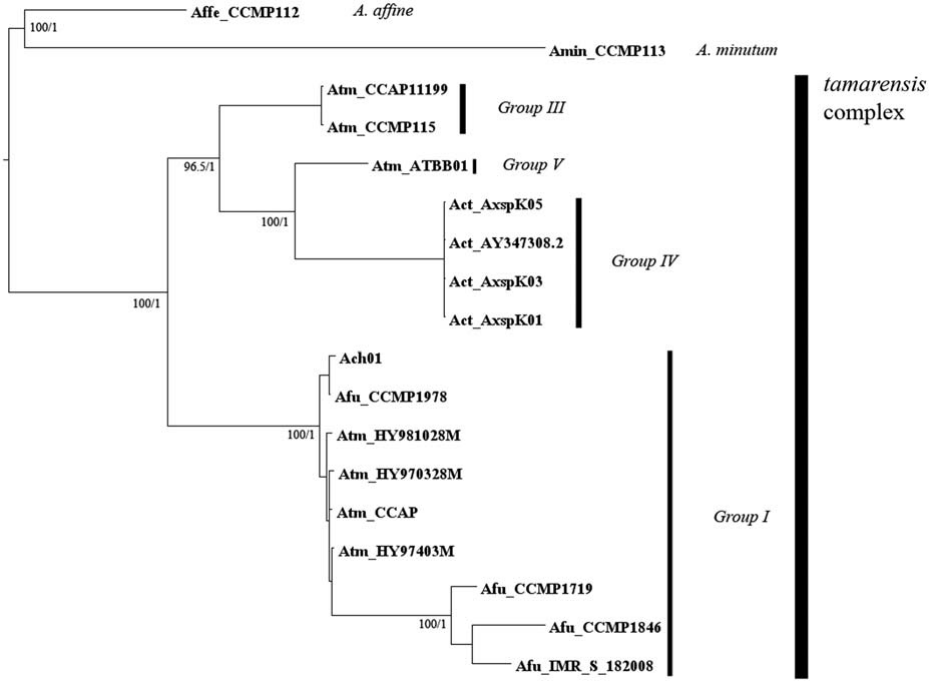
**Phylogenetic tree of 17 *Alexandrium* species and strains (Atm, *A. tamarense*; Act, *A. catenella*; Afu, *A. fundyense*; Amn, *A. minutum*; Aaf, *A. affine*) and local *Alexandrium* species Ach01.** Sequences were obtained from the GenBank database using the keywords ‘Alexandrium LSU rDNA’ or ‘Alexandrium 28S’. Phylogenetic trees were generated for an alignment of 18 sequences of 1776 bp in the ITS1-D5 region through Bayesian inference in MrBayes V3.2 and maximum likelihood (ML) in PhyML. Bayesian analysis was carried out with 1 500 000 runs, with five separate initial trees. Convergence was checked through PSRF and AVSF. FigTree V1.3.1 was used as a visual representation of output trees. For ML analysis these were carried out with 1000 bootstrap replicates.

In order to achieve further resolution of the *tamarensis* complex, 18 sequences longer than 1776 bp, from different species and strains of the *tamarensis* complex, located in the rDNA region were analysed (Fig. 4). The total alignment involved ITS1, 5.8S rDNA, ITS2 and 1185-bp of the D1–D5 regions of the 28S rDNA (Ki and Han 2007). Sequences of *A. minutum* and *A. affine* were selected as outgroup species for the *tamarensis* complex.

For maximum likelihood analysis, the logarithm of the likelihood score of the optimal tree was −6591.25984. As expected, the results were again consistent and the local *Alexandrium* strain was allocated to Group I of the *tamarensis* complex.

Topologies for trees generated through maximum likelihood and Bayesian inference were the same, and had high bootstrap and posterior probability values (Figs 3 and 4). Group generation was carried out analysing clades formation and the previous literature. Comparing the structure of both trees, using 18 or 81 sequence alignments, the same clades were formed.

In order to obtain information on the variability of the amplified region for both data sets, the average number of differences per pair of sequences aligned and the number of parsimonious sites were measured. For the first data set, values were 8.37 bp and 130, respectively. On the other hand, for the second data set, the average number of differences per pair of sequences aligned was 52.03 bp and the number of parsimonious sites was 298. Considering the 1185-bp segment covering only the LSU rDNA region, the amount of parsimonious informative sites and the average number of differences per pair of sequences aligned decreases from 298 to 174 and from 52.02 to 35 bp, representing a fall of 42 and 32 %, respectively, with respect to the whole amplified region.

### *Alexandrium* DNA detection in challenged *Mytilus* samples by real-time PCR

From each aquarium, DNA from the gill tissues of five different randomly picked *Mytilus* challenged *in vivo* with *Alexandrium* strain ACC02 were used to detect the presence of *A. tamarense* through a real-time PCR assay with species-specific primers. We obtained similar and positive results for the samples, which were extracted between Days 2 and 5 of the toxicfeeding phase, with Ct values ranging from 21 to 22. On the other hand, all samples extracted on Day 15 of the detoxification phase gave no specific amplification, indicating that there was no detectable *Alexadrium* DNA in the *Mytilus* samples (Table 3). Replicates of *Mytilus* samples challenged in parallel in four independent aquaria showed the same pattern, confirming the detection of *Alexandrium* in *Mytilus* gills during the toxic phase. Through amplicon melt analysis and direct sequencing of the 235-bp PCR-amplified fragment, the region corresponded to the expected specific sequence within the D1/D2 LSU rDNA domain, according to Hosoi-Tanabe and Sako (2005).

**Table 3:** Detection of *Alexandrium* DNA in gill tissue samples from challenged *Mytilus* through days 2 to 27 by q-PCR.

Sample	Challenge periods (days)	Ct
1	2^a^	21.0
2	3^a^	21.2
3	4^a^	21.1
4	5^a^	22.0
5	15^b^	NA

*Mytilus* were exposed to a contaminated diet (1.7–2.0 mg L^−1^; dry weight) containing 50 % toxic dinoflagellate *Alexandrium* strain ACC02 and 50 % *I. galbana* (by weight) for a period of 12 days, followed by a detoxification period of 15 days, where they were fed with *I. galbana*. Animals were dissected alive on Days 2, 3, 4 and 5 of the toxic phase and Day 15 of the detoxification phase, and 30 g of drained gill tissue were used as the starting material for DNA purification with the DNeasy Blood & Tissue Kit (Qiagen) according to the manufacturer's protocol. Real-time PCR assays using species-specific *A. tamarense* primers were carried out in extracted DNA from *Mytilus* in order to determine the possibility of detecting *Alexandrium* DNA in challenged mussel tissue. NA, no amplification.

^a^Days in toxic phase.

^b^Days in detoxification phase.

## Discussion

The study of HABs has become increasingly important given the recent rise in the number and frequency of toxic events, and associated adverse impacts on public health, fisheries and ecosystem services (Anderson 1989; Cassis *et al*. 2002). This threat has led to extensive investigation on how to develop international standards for the detection of toxins in seafood and on the implementation of expanded monitoring programmes for toxic algae. One of the most frequently used methods for the evaluation of toxins in HAB episodes worldwide has been the mouse bioassay. Unfortunately, this technique does not always provide accurate estimates of toxicity (Fernández *et al*. 2002) and requires considerable resources and time. Moreover, this technique has been questioned with regard to animal welfare and is prohibited in some countries. In this context, increasing efforts have been focused on monitoring the toxic algae directly to predict their occurrence and better allocate sampling effort, particularly with regard to toxin analysis.

In this paper, we present phylogenetic analyses using ITS1-D5 rDNA sequence data which demonstrate that the Chilean strains analysed belong to Group I in the *tamarensis* complex, rather than to the *A. catenella* grouping (Scholin *et al.* 1994; Medlin *et al.* 1998; Higman *et al.* 2001; Lilly *et al.* 2007). The first studies concerning Chilean HABs carried out 40 years ago, based on morphological observations, identified *A. catenella* as the dominant *Alexandrium* species (Muñoz 1985). This identification has never been questioned or assessed by more accurate methods such as genomic sequencing. The phylogenetic analysis carried out in this study clearly indicates that the local *Alexandrium* species belongs to the Group I ribotype. This group is mainly composed of *A. tamarense*, in contrast to Group IV in which the predominant species is *A. catenella*. Similarly, phylogenetic trees based on *Alexandrium* toxin variability showed that strains from Argentina, Brazil, Chile and Uruguay belonged to the same clade, paralleling the Group I results (Montoya *et al.* 2010)*.* As red tide blooms have been present since the 19th century in Chile and Brazilian, Uruguayan and Argentinian events are more recent, it has been proposed that toxic episodes in Eastern South America could be due to the expansion of Chilean *Alexandrium* species (Lilly *et al.* 2007). Even more, Uruguayan and Brazilian strains have been classified as *A. tamarense* in Group I, consistent with our findings in relation to the fact that Chilean species, supporting the hypothesis that the local strain was missclassified as *A. catenella*.

Very recently, Miranda *et al.* (2012) discussed the validity of using direct amplification sequences of the rDNA subunits as a suitable method for strain differentiation in *Alexandrium* species, due to the existence of paralogue genes. In this respect, base quality discrimination did not give evidence of paralogue sequences. On the other hand, Ki and Han (2007), eliminating paralogue sequences for their analysis, found 39 parsimony informative sites within the D1–D5 LSU rDNA region in five different *Alexandrium* species. Our study found a much higher value of parsimony variable sites (174) when our local sequence was aligned with published sequences, which probably contained paralogue DNA regions. It is unlikely that this considerable difference could be explained by increased mutation rates in the sequenced regions. Thus, this result agrees with Miranda *et al.* (2012), who suggest that the great diversity in Group I of the *tamarensis* complex could be due to the lack of an accurate discrimination of paralogue sequences.

The sequence analysis facilitated the use of a specific and highly sensitive real-time PCR assay to detect local *Alexandrium*. Owing to the ability of filter-feeding molluscs to capture and concentrate phytoplankton, by pumping water through their gill filaments, we tested the possibility of detecting dinoflagelate DNA in this organ of challenged mussels. Preliminary experimental results showed, for the first time, the implementation of a practical test to detect these algae in gill tissue extracted from mussels challenged under laboratory conditions. Currently, we are working on the implementation of this test in field samples in order to detect traces of *Alexandrium* in seawater. It would be very useful to count with methods to detect traces of dinoflagellates, in order to predict massive algal blooming through constant monitoring of red tide episodes, thus preventing human consumption of toxic filter-feeding shellfish.

## Conclusions and forward look

Traditionally, South American Pacific HABs have been assigned to *A. catenella*, based only on morphological evidence that has proven to be an unreliable indicator of species identification within the *A. tamarense* complex (Lilly *et al.* 2007).

This study was focused on the molecular identification of the *Alexandrium* species that causes paralytic shellfish poisoning in Chilean coasts (Hernández *et al.* 2005). Phylogenetic analyses based on sequence data and species-specific PCR assays targeting LSU rDNA and microsatellite regions, all demonstrate that cultures isolated from Chilean coasts belong to the *tamarensis* complex Group I and are not *A. catenella* (Figs 1 and 2, Tables 1 and 2, Hosoi-Tanabe and Sako 2005).

As not all *A. tamarense* are toxic, we are currently developing a real-time PCR assay based on primer pairs that target signature nucleic acid sequences of genes involved in toxin production. Our goal is to set up a new technique for early, sensitive and accessible HAB detection in order to avoid the important financial damage and public health issues.

## Additional information

The following additional information is available in the online version of this article –

Sequences used for phylogenetic analyses.

File 1: DNA sequences used in this study. Strain assignment, morphospecies, origin and accession number in GenBank are given when available.

## Accession numbers

The Ach01 (NCBI accession no. JN657223) sequence was uploaded to GenBank.

## Sources of funding

This work was funded by the Corporación del fomento de la producción (Corfo) of Chile through the INNOVA Chile Project #07CN13PPD-240.

## Contributions by the authors

A.J. and G.F. did the sequence analysis, phylogenetic analysis, PCR experiments and writing of the manuscript. M.A., P.O., J.E.T. and J.M.N. contributed with the challenge of the *Mytilus* spp. samples with local *Alexandrium* species. V.M. developed the idea, provided further inputs during the project and contributed to the financial means for carrying out the project.
